# Quantum coherence selective 2D Raman–2D electronic spectroscopy

**DOI:** 10.1038/ncomms14732

**Published:** 2017-03-10

**Authors:** Austin P. Spencer, William O. Hutson, Elad Harel

**Affiliations:** 1 Department of Chemistry, Northwestern University, 2145 Sheridan Road, Evanston, Illinois 60208, USA

## Abstract

Electronic and vibrational correlations report on the dynamics and structure of molecular species, yet revealing these correlations experimentally has proved extremely challenging. Here, we demonstrate a method that probes correlations between states within the vibrational and electronic manifold with quantum coherence selectivity. Specifically, we measure a fully coherent four-dimensional spectrum which simultaneously encodes vibrational–vibrational, electronic–vibrational and electronic–electronic interactions. By combining near-impulsive resonant and non-resonant excitation, the desired fifth-order signal of a complex organic molecule in solution is measured free of unwanted lower-order contamination. A critical feature of this method is electronic and vibrational frequency resolution, enabling isolation and assignment of individual quantum coherence pathways. The vibronic structure of the system is then revealed within an otherwise broad and featureless 2D electronic spectrum. This method is suited for studying elusive quantum effects in which electronic transitions strongly couple to phonons and vibrations, such as energy transfer in photosynthetic pigment–protein complexes.

## Abstract (web summary)

Electronic and vibrational correlations report on the dynamics and structure of molecular species, yet revealing these correlations experimentally is challenging. Here the authors develop a method called GAMERS that probes correlations between states within the vibrational and electronic manifold with quantum coherence selectivity.

The interaction between different degrees of freedom in coupled molecular systems—electrons, nuclei and phonons—dictate many of their most important physical properties. For instance, electron–phonon interactions underlie the carrier properties of a wide range of promising light harvesting materials including hybrid perovskites^1^,^2^, organic crystals that undergo singlet fission^3^,^4^ and quantum-confined nanostructures^5^,^6^,^7^,^8^. In natural systems, interactions between electronic and vibrational degrees of freedom may have important implications for rapid and efficient energy transfer^9^,^10^,^11^,^12^,^13^,^14^. Despite tremendous effort, revealing these interactions experimentally has proved extremely challenging. For instance, the physical origins of some coherent phenomena observed^15^,^16^,^17^ in photosynthetic pigment–protein complexes using two-dimensional Fourier-transform (2DFT) spectroscopy remain elusive, partly due to the ambiguity of spectral signatures arising from related signal pathways. Although much progress has been made in better understanding and modelling these protein complexes^13^,^14^,^18^,^19^,^20^,^21^,^22^, until now there has been no direct (that is, model independent) method for assigning these signals to specific coherent electronic or vibrational pathways, therefore leaving their physical origins unresolved.

The shortcomings of current four-wave mixing (4WM) techniques, such as 2DFT spectroscopy^23^,^24^, motivate the development of higher-order spectroscopies that may enable unambiguous assignment of signals to specific coherent pathways. In this work, we present an approach that provides direct correlation between impulsively driven low-frequency modes such as phonons, vibrations and (multi-)excitons with quantum coherence selectivity through control of resonance. This six-wave mixing (6WM) Raman–electronic spectroscopy produces a fully coherent 4D correlation spectrum between select ground and excited state vibrations and ground and excited state electronic transitions. In essence, this technique generates a 2D Raman-like spectrum for each point in the 2DFT electronic spectrum. Among the many unexplored physical phenomena accessible by this technique, we demonstrate the ability to isolate signals from—and assign signals to—distinct vibronic pathways and to spectroscopically distinguish ground and excited state vibrational coherences. We call this method gradient-assisted multidimensional electronic Raman spectroscopy (GAMERS). The previously described 2D Resonance Raman techniques^25^,^26^ share similarities with this method, but—among other important distinctions—yield only a subset of the information contained in a GAMERS spectrum. Critically, we establish that GAMERS measurements are free of the lower-order cascades^25^,^27^,^28^ that have hindered prior non-resonant 2D Raman techniques^29^,^30^,^31^. Specifically, we show that GAMERS directly measures the coupling of low-frequency vibrational modes to one another as well as to higher-frequency electronic transitions in an organic dye in dilute solution.

## Results

### Methodology

**Figure 1:**
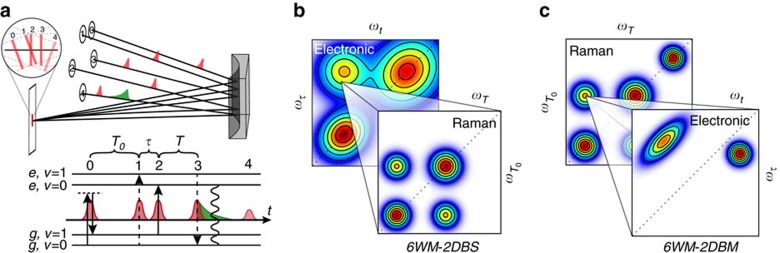
Overview of the GAMERS technique. (**a**) In a simplified schematic of gradient-assisted multidimensional electronic Raman spectroscopy (GAMERS), 5 pulses are focused to a line in the sample and the radiated signal field is spectrally dispersed onto a 2D CMOS sensor (camera), where it is detected as a function of wavelength (horizontal dimension) and the delay *τ* (vertical dimension). An inset shows the pulse tilts at the focus. Below, an energy ladder diagram depicts an example six-wave mixing (6WM) signal generation pathway in a four-level system composed of two electronic states (*g* and *e*), each with two vibrational levels (*v*=0 and *v*=1). (**b**) Slices of a model 4D 6WM spectrum illustrate that it effectively contains a 2D Raman spectrum (6WM 2D beating slice spectrum) at each point in the 2D electronic spectrum. (**c**) A beating map (6WM 2D beating map spectrum) constructed from a specific 2D Raman frequency highlights parts of the 2D electronic spectrum associated with the chosen beating pathway.

The GAMERS apparatus (Fig. 1a) capitalizes on a method recently introduced called GRadient-Assisted Photon Echo Spectroscopy (GRAPES)^32^, which captures a complete 2DFT spectrum in a single laser shot. Building on top of the GRAPES concept to exploit spatial multiplexing is necessary here since including an additional time–frequency dimension would otherwise require scanning three independent time axes, making the experiment intractable (Supplementary Note 1). In GAMERS, an additional pulse serves to excite the sample into a non-equilibrium state before being interrogated by the GRAPES pulse sequence. This pre-pump pulse (denoted pulse 0) contributes two field–matter interactions and is followed by three excitation pulses (pulses 1, 2 and 3), each contributing a single interaction, thereby generating a fifth-order, 6WM signal.

In a typical four-wave mixing (4WM) process on a two-level system, pulse 1 excites a single-quantum coherence (SQC), pulse 2 converts the SQC into a zero-quantum coherence (ZQC) and pulse 3 reforms a SQC. The SQC represents a superposition of electronic states, while the ZQC represents a wave packet within either the ground or excited electronic state manifolds. In GAMERS, pulse 0 effectively excites a ZQC on the ground electronic state, producing a non-thermally equilibrated initial state (that is, a perturbed density matrix) that is probed by the subsequent three-pulse sequence. Altogether, the four excitation pulses generate a fifth-order signal in the phase-matched directions given by 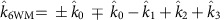, which effectively coincides with the third-order GRAPES signal since the two oppositely-signed 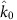 terms approximately cancel. The desired 6WM signal is isolated from collinear 4WM signals using synchronous detection while amplitude modulating the pre-pump pulse and pulse 3. Although, there are disadvantages to using a non–background-free geometry, one notable advantage is that this approach enables the 6WM signal to be heterodyned with the 4WM signal to determine its relative phase, providing a convenient estimate of the influence of undesired third-order cascaded signals. Previously, we analysed the concentration dependence to insure that 6WM signal generated using a 1,028 nm pre-pump was not dominated by cascades^33^. Here, we explicitly examined the relative phase between the 4WM and 6WM signals. The two additional interactions involved in 6WM introduce a *π* phase shift relative to 4WM (refs 25, 26), a result that is borne out in the 6WM signal as a negative amplitude when heterodyned with 4WM. Conversely, cascaded third-order signal is expected to be in-phase with the 4WM signal, thus producing a positive amplitude^25^,^26^.

The signal in GAMERS is acquired as a function of four independent time–frequency dimensions (Fig. 1a): the delays between pulses 0 and 1 (*T*_0_), 1 and 2 (*τ*), and 2 and 3 (*T*) and the frequency of the emitted signal (*ω*_*t*_). The complete frequency and coherence time (*τ*) dimensions are acquired simultaneously for each laser shot by spatially encoding *τ* in the sample using pulse tilts with the GRAPES geometry (see inset of Fig. 1a)^32^. This is accomplished by focusing all the beams using a cylinder mirror and tilting the pulse fronts using plane mirrors. Judicious choice of the beam geometry generates a spatial encoding of the time delays across the unfocused spatial direction. GRAPES typically encodes the *τ* delay, although in principle other delays could be encoded by changing the phase-matching geometry. Since *ω*_*t*_ is measured directly by a spectrometer, only *T*_0_ and *T* need to be explicitly scanned. Further, the phase-matched geometry must be carefully designed such that no temporal gradient between pulses 0 and 1 is introduced, ensuring that *T*_0_ stays constant across the spatial axis of the sample. The multiplex advantage afforded by the GRAPES geometry enables acquisition of a large 4D space (∼6.5 × 10^8^ distinct points), which would otherwise take weeks to fully sample. While the system is passively phase stabilized, the maximum measurement window is typically only several hours with five beams (Raman pre-pulse, three excitation beams and a local oscillator), precluding any attempts to perform the experiment in the traditional point-by-point fashion.

The 4D signal is then Fourier-transformed along the three time dimensions to generate a 4D GAMERS spectrum, 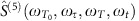. To view and interpret the 4D GAMERS spectrum, 2D slices of the data are extracted and processed into three new types of 2DFT spectra. A 6WM 2DFT electronic slice (6WM-2DES) spectrum shows the correlation between electronic transitions and is generated by taking a slice of the 6WM signal 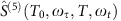 at a chosen (*T*_0_, *T*) location. 6WM 2DFT beating slice (6WM-2DBS) spectra exposes correlation between vibrational transitions and their coupling to specific electronic transitions. A 6WM-2DBS spectrum is generated from 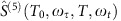 by choosing a specific (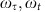) location, removing the influence of population dynamics using a multi-exponential model, and Fourier transforming along *T*_0_ and *T*. As an example, Fig. 1b shows a model 6WM-2DES spectrum (labelled ‘electronic') and an accompanying 6WM-2DBS spectrum (labelled ‘Raman'). Last, a 6WM 2DFT beating map (6WM-2DBM) spectrum is constructed by taking a slice of 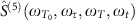 at a chosen 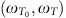 location. As illustrated in Fig. 1c, the 6WM-2DBM spectrum highlights locations in the 2DFT electronic spectrum that contain a specific 2D beating frequency.

GAMERS shares similarities with a set of techniques referred to collectively as 2D Resonance Raman (2DRR) spectroscopy^34^. The FSRS-like approach to 2DRR (ref. 35) is a mixed time–frequency domain method in which the *ω*_*T*_ dimension is directly detected on a spectrograph as a function of *T*_0_ at a fixed *τ* using narrowband Raman pump pulses. This approach yields a 2D Raman spectrum 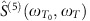 upon Fourier transforming *T*. The all-broadband approach to 2DRR (ref. 25) uses only broadband pulses, relying on Fourier transforms for frequency resolution. In this method, the broadband signal field is detected as a function of *T*_0_ and *T* using spectral interferometry to produce a 3D spectrum 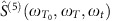 with two Raman frequency dimensions and one directly detected electronic frequency dimension. The implications of using different detection methods and excitation pulse resonance conditions are further addressed in the Discussion.

### Pathway selectivity

The spectrum of the Raman pre-pump is an essential design choice, since it determines which electronic states can be populated during the *T*_0_ time interval. We utilized an 850 nm wavelength pre-pump, which is red-shifted relative to the lowest electronic transitions of IR-140, the cyanine dye studied here. (Additional measurements on IR-140, IR-895 and IR-144 utilizing a 1,028 nm pre-pump are presented in Supplementary Note 2; Supplementary Figs 1–6.) This pre-resonant condition guarantees that only ground state vibrational wavepackets are formed during *T*_0_, excluding all pathways involving excited state vibrational and electronic coherences. Unlike other mixed time–frequency 6WM methods, the use of a pre-resonant pump in GAMERS greatly simplifies the resultant spectra by reducing the number of detected signal pathways. This electronic state specificity enables coherences in the *T*_0_ and *T* periods to be assigned to specific pathways.

Figure 1a (lower half) illustrates an energy ladder diagram^36^ (Supplementary Note 3) for one of several 6 WM signal pathways involving a four-level system composed of two electronic states, each with two vibrational levels. In this pathway, the non-resonant pre-pump pulse excites a coherent superposition of vibrational states (that is, vibrational coherence) on the ground electronic state. This vibrational coherence oscillates at the 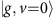
→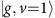 transition frequency during the *T*_0_ interval. Subsequent interactions with pulses 1 and 2 promote the molecule to a vibrational coherence in the excited electronic state, which oscillates at the vibrational difference frequency between 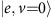 and 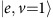 during *T*. Finally, pulse 3 generates an electronic coherence which ultimately radiates the 6WM signal. This pathway would produce a cross peak in the 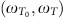 projection of the GAMERS spectrum, indicating that these vibrations are coupled through a common set of electronic states.

An important concern in fifth-order spectroscopies is the potential influence of cascaded lower-order signals. A number of tests were undertaken in order to determine the prevalence of cascades in our measurements. First, it was confirmed that the 6WM signal depends linearly on chromophore concentration at low concentration, and not quadratically as expected for cascaded pathways^25^,^28^,^31^,^37^. While this places an upper bound on the influence of cascades involving only solute molecules, it does not preclude pathways where a 4WM signal radiated by a solvent molecule serves as an excitation field for 4WM in a solute molecule. Such a solvent–solute cascade signal would depend linearly on the concentrations of the solvent and the solute. The viability of this type of cascade was tested by measuring the spectrally-resolved transient absorption of the solvent using a pre-resonant pump and a resonant 780 nm probe, which simulates the first 4WM step of the solvent–solute cascade, and comparing to the same transient absorption measurements taken on IR-895, IR-140 and IR-144 solutions. The signal size for the neat solvent was 200 to 4,000 times smaller than for the dye samples, supporting the conclusion that cascade pathways involving the solvent do not have a significant influence on the 6WM measurements.

### Raman-electronic spectra of IR-140

**Figure 2:**
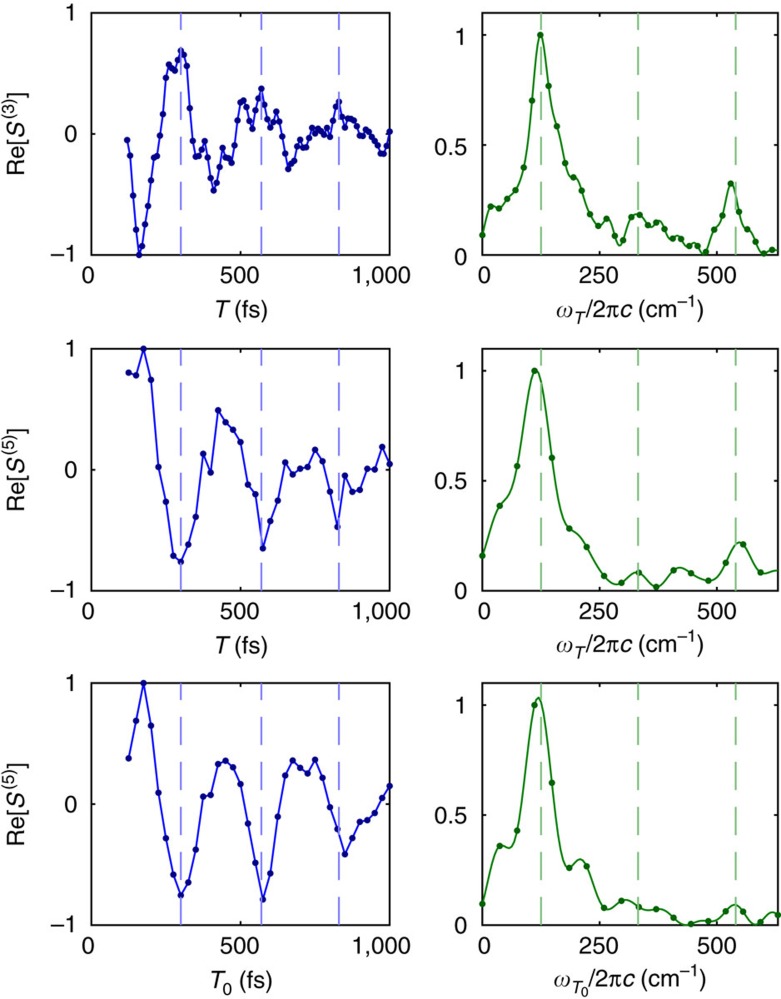
Comparison of the oscillatory part of the 4WM and 6WM signals in IR-140. The top row of panels show the real part of the four-wave mixing (4WM) signal as a function of *T* (left panel) and its Fourier transform (right panel). The middle and bottom rows of panels show the real part of the six-wave mixing (6WM) signal as a function of *T* at *T*_0_=100 fs and as a function of *T*_0_ at *T*=100 fs, respectively, along with their Fourier transforms. All transients were taken from the blue side (along *ω*_*t*_) of the main peak in the 6WM 2DFT electronic spectrum. Blue dashed lines mark peaks (troughs) in the 4WM (6WM) transients. Green dashed lines mark the 125, 330 and 540 cm^−1^ oscillation frequencies.

4WM 2DFT spectra were collected and used as a baseline for comparison to the 6WM GAMERS spectra, providing information on the amplitudes and frequencies of accessible Raman-active modes. Figure 2 demonstrates some of the similar features observed in 4WM *T* transients when compared to 6WM *T* and *T*_0_ transients. Each transient contains a strong 125 cm^−1^ oscillation along with weaker oscillations at 330 and 540 cm^−1^, as exemplified by the peaks in their respective spectra. The low-frequency 125 cm^−1^ oscillation visible in the transients exhibits a clear π phase shift between the 4WM and 6WM signals, as expected for signals of the form 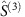
∝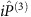 and 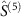
∝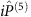, respectively^25^,^26^. This phase shift demonstrates that GAMERS successfully isolates 6WM signal from other pathways, including cascades.

**Figure 3:**
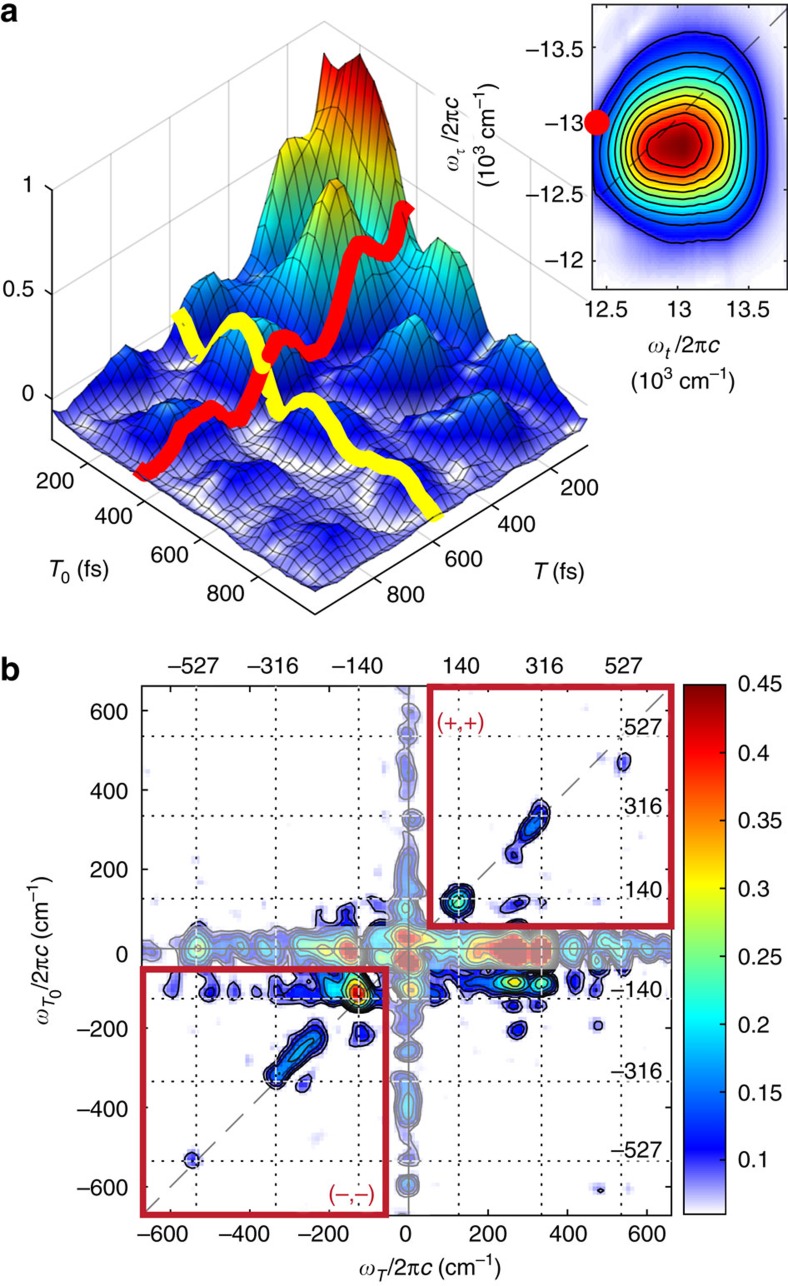
Beating slices taken from the 6WM 4D GAMERS spectrum of IR-140. From the Gradient-Assisted Multidimensional Electronic Raman Spectrum (GAMERS), the top panel (**a**) shows the real-valued six-wave mixing (6WM) 2D transient taken at 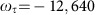 cm^−1^ and 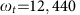 cm^−1^, indicated by the red dot in the absolute value 6WM 2DFT electronic spectrum for *T*_0_=0 and *T*=100 fs (inset). Red and yellow lines highlight oscillations along the *T* and *T*_0_ dimensions, respectively. (**b**) A 6WM 2D beating slice spectrum exhibits Raman vibrations on the diagonal primarily in the upper right (+, +) and lower left (−, −) quadrants at ±140, ±316 and ±527 cm^−1^.

Figure 3a presents a 6WM-2DES spectrum of IR-140 and a real-valued 2D 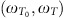 transient taken from a single 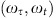 location in the spectrum (indicated by a red dot). The 6WM-2DES spectrum of IR-140 closely resembles the 4WM 2DFT electronic spectrum at comparable *T* delays. The 6WM transient exhibits damped oscillatory components arising from coherent vibrational beating during both *T*_0_ and *T*. Oscillations that evolve in phase in the *T*_0_+*T* direction result in diagonal peaks when Fourier transformed along *T*_0_ and *T* to form the 6WM-2DBS spectrum in Fig. 3b.

### Spectral correlations

A 6WM 2D beating slice (6WM-2DBS) spectrum of IR-140, presented in Fig. 3b, contains a range of features including diagonal peaks (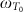
=*ω*_*T*_), cross peaks (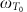
≠*ω*_*T*_) and zero-frequency peaks (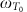
=0 and/or *ω*_*T*_=0). Diagonal peaks expose the correlation between equivalent coherences during the *T*_0_ and *T* time intervals while cross peaks indicate coupling between different coherences. Zero-frequency peaks indicate that a given coherence during either *T*_0_ or *T* couples to a non-oscillatory population in the other time interval. Since only ground state vibrational coherence pathways are excited during *T*_0_, the 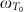 frequencies for the set of peaks observed at *ω*_*T*_=0 and the diagonal must correspond to ground state vibrations. The additional frequencies observed along *ω*_*T*_—but not 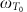
—can be assigned to vibrational modes of the excited state.

6WM-2DBS spectra of IR-140 exhibit peaks primarily in the lower left (−, −) and upper right (+, +) quadrants of 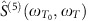. The (+, −) and (−, +) quadrants are mostly empty because they involve transitions with either weak transition dipole moments or poor overlap with the laser spectrum. For instance, the pre-pump pulse may create a 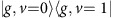 coherence in the *T*_0_ period, but then would require a 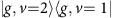 coherence during *T* to show up in the (+, −) quadrant. The intermediate electronic transition 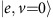
→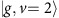 is likely redshifted relative to the pulse spectrum, weakening it compared to the transitions present in the (+, +) and (−, −) quadrants. While the frequencies and amplitudes of vibrations in the (+, +) and (−, −) quadrants nearly mirror one another, they arise from distinct coherence pathways. The peaks that appear in the (−, −) quadrant involve vibrational coherences with negative frequencies during *T*_0_ and *T*, while those in the (+, +) quadrant are associated with positive frequencies. The sign of these coherences influences subsequent field-matter interactions, resulting in signal pathways whose peak shapes and frequencies along the electronic dimensions 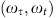 are distinct for (−, −) and (+, +) vibrational coherences. The coherence pathway specificity afforded by GAMERS therefore enables the sub-structure of the electronic spectrum to be dissected. By separating signals based on their distinct low-frequency coherences, the broad vibronic pathways that otherwise overlap spectrally in conventional 4WM 2DFT spectroscopy are isolated, revealing sharper features underneath the electronic transition band. This capability is demonstrated below.

**Figure 4:**
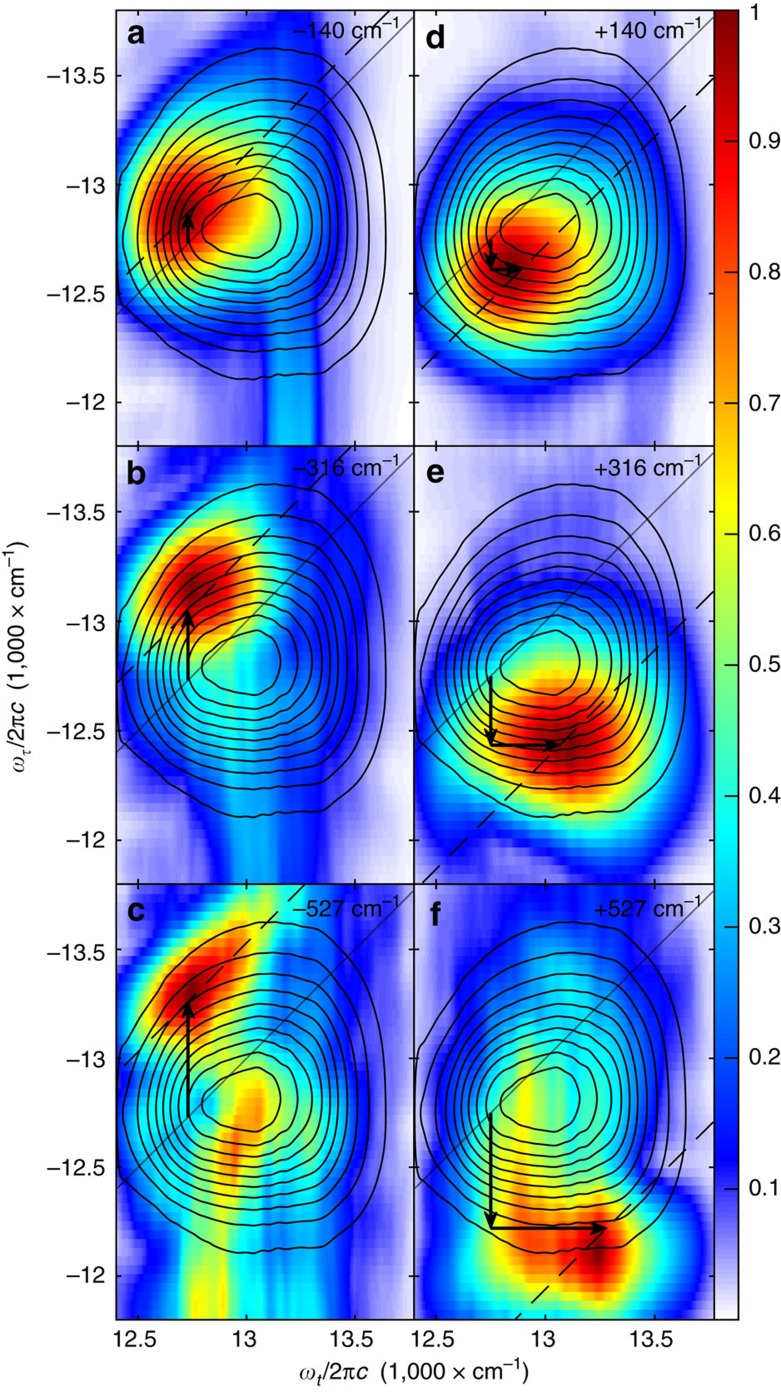
Beating signatures in the 6WM GAMERS spectrum of IR-140. Beating maps are constructed from diagonal peaks (that is, 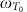
=*ω*_*T*_) at (**a**) −140 cm^−1^, (**b**) −316 cm^−1^, (**c**) −527 cm^−1^, (**d**) 140 cm^−1^, (**e**) 316 cm^−1^ and (**f**) 527 cm^−1^ in the 4D Gradient-Assisted Multidimensional Electronic Raman spectrum (GAMERS). A contour plot of the absolute value six-wave mixing 2D electronic slice (6WM-2DES) spectrum at *T*_0_=0 and *T*=100 fs is overlaid for reference. Dashed lines are displaced from the diagonal (solid line) by the 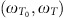 beating frequency along (**d**–**f**) the *ω*_*τ*_ and *ω*_*t*_ dimensions or (**a**–**c**) the *ω*_*τ*_ dimension only.

6WM 2D beating map (6WM-2DBM) spectra of IR-140, presented in Fig. 4a–c, were generated for the three negative beating frequencies on the 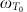
=*ω*_*T*_ diagonal marked by dotted lines in Fig. 3b. These frequencies correspond to beating modes observed in 4WM 2DFT electronic spectra as a function of *T*, and are the strongest diagonal features in the 6WM-2DBS spectrum. Compared to the 6WM-2DES spectrum (overlaid as contour lines in Fig. 4), the beating maps differ in peak shape and are overall redshifted along *ω*_*t*_. It has been reported that wavepacket beating is typically strongest when probing near the classical turning points of the potential energy well^38^, an effect that may contribute to this *ω*_*t*_ redshift. The main peak in the beating maps also exhibits a shift relative to the diagonal that depends on the beating frequency. This shift occurs primarily along *ω*_*τ*_ and is equal to the (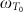,*ω*_*T*_) frequency from which the corresponding beating map is constructed (marked with a dashed line for visual reference). 6WM-2DBM spectra of IR-140 generated for positive beating frequencies, shown in Fig. 4d–f, also exhibit peak shifts dependent on the chosen (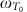,*ω*_*T*_) frequency. Although similar to the peak shifts for (−, −) quadrant beats, beats in the (+, +) quadrant are distinct in that the electronic peak shifts occur along both *ω*_*τ*_ and *ω*_*t*_ due to differences in accessible coherence pathways.

## Discussion

**Figure 5:**
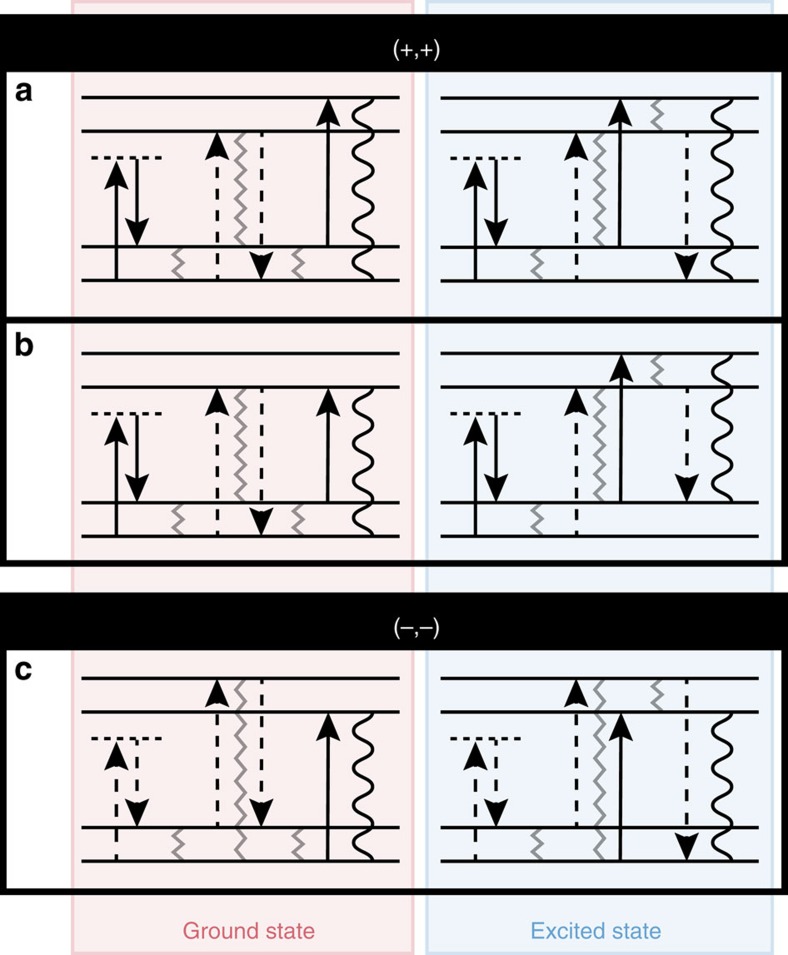
Coherence pathways involving correlated Raman vibrations. Energy ladder diagrams (that is, wave mixing diagrams) depict vibrational coherence pathways that yield the diagonal Raman peaks in (**a**,**b**) the (+, +) quadrant and (**c**) the (−, −) quadrant of the six-wave mixing 2D beating slice (6WM-2DBS) spectrum (Fig. 3b). These pathways are consistent with the Raman frequency–dependent peak shifts observed in the six-wave mixing beating map (6WM-2DBM) spectra (Fig. 4). Grey, zig-zag lines mark coherently linked vibronic states during *T*_0_, *τ* and *T*, and a black wavy line represents the final electronic coherence that radiates the signal during *t*.

Correlations between the vibrational and electronic frequencies of peaks in the GAMERS spectrum arise from signal generation pathways wherein the initial ground state vibrational coherence frequency in *T*_0_ influences the electronic coherence frequency during *τ* and/or *t*. Figure 5 enumerates several such pathways, categorizing them based on the sign of their vibrational coherence frequency, as well as their electronic state character (excited versus ground state) during *T*. The positive vibrational frequency pathways in Fig. 5a are responsible for generating frequency-shifted peaks in the 6WM-2DBM spectra of Fig. 4d–f. The coherence frequency during *T*_0_ subtracts from the subsequent electronic coherence during *τ*, inducing a red shift along *ω*_*τ*_. After the second field interaction, the vibrational coherence is either reformed on the ground electronic state or transferred to the excited state, oscillating with a positive frequency during *T*. As opposed to subtracting from the electronic coherence like in *ω*_*τ*_, here the vibrational coherence frequency adds to the electronic coherence generated by the third field interaction, blue shifting the radiated signal field along *ω*_*t*_. The opposing shift in *ω*_*τ*_ and *ω*_*t*_ are due to the opposite signs of the first and third interactions. For the 6WM-2DBM spectrum of the 527 cm^−1^ vibrational frequency (Fig. 4f), broadening of the peak shape along *ω*_*t*_ is evident and suggests the presence of an additional pathway that is red shifted only along *ω*_*τ*_. This peak arises from the pathways in Fig. 5b, which generate signal through a radiation step that conserves the vibrational state (that is, *δv*=0). These pathways are likely weaker due to reduced overlap between the laser spectrum and the 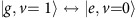 transition. The vibrational peaks in the (−, −) quadrant of the 6WM-2DBS spectrum are attributed to the pathways shown in Fig. 5c, which involve a negative frequency coherence during *T*_0_ that blue shifts the subsequent electronic coherence during *τ* but does not influence the detection frequency *ω*_*t*_. The strongest peaks visible in the 6WM-2DBS spectrum lie directly on the diagonal, which is strongly suggestive of an exclusively ground-state origin. A vibration on the excited electronic surface would be expected to be somewhat shifted along *ω*_*T*_ from that on the ground electronic state due to differences in curvature of the nuclear potential energy surfaces of the ground and excited electronic states; this signature is not observed in the 6WM-2DBS spectrum.

GAMERS has several notable differences in information content when compared to related 2DRR techniques^34^. 2DRR methods employing only resonant excitation pulses generate 2D Raman spectra that, in general, contain both ground and excited electronic state vibrational pathways. By contrast, GAMERS utilizes pre-resonant excitation to isolate ground and excited state vibrational pathways. In addition, GAMERS is a fully Fourier transform method that achieves molecule-limited resolution along all four dimension. On the other hand, the FSRS-like approach to 2DRR is a mixed time–frequency domain technique which complicates the analysis of spectral dynamics and places restrictions on achievable time resolution^39^. These limitations are a consequence of direct detection of one Raman vibrational dimension, eliminating the need to scan the final population time (*T*). Ultimately, FSRS-like 2DRR yields 2D Raman spectra without resolved electronic frequency dimensions, eliminating information related to electronic–vibrational and electronic–electronic coupling.

The all-broadband approach to 2DRR avoids the limitations of the mixed domain methodology but, by fixing *τ*=0, also lacks the electronic excitation dimension (*ω*_*τ*_) that is acquired in GAMERS in a single laser shot through spatial multiplexing without the cost of additional delay scanning. The one-dimensional electronic nature of all-broadband 2DRR masks the rich variations between two-dimensional electronic peak shapes associated with distinct 2D Raman peaks. Some 6WM-2DBM spectra, like those in Fig. 4a–c, exhibit differences in peak location only along the *ω*_*τ*_ dimension, a feature that would be hidden in 2DRR. Without frequency resolution along *ω*_*τ*_ and *ω*_*t*_, the advanced coherence pathway analysis demonstrated here would not be possible.

Through analysis of 6WM-2DBM spectra, we have demonstrated the capability of GAMERS to provide vibrational resolution within very broad (>700 cm^−1^) room temperature electronic peak shapes. This ability is significant in that it enables, for example, overlapping electronic dynamics from distinct sub-units in a chemical complex to be isolated based on their coupling to different vibrational modes. Vibrational resolution provides a powerful method to untangle independent signal pathways in spectrally congested systems and to probe local structure through vibrational coupling. With further refinements to minimize the influence of scattered excitation light and maximize signal collection, this technique is poised to tackle complex biological systems in which electronic and vibrational correlation information will play a key role in deciphering their functional mechanisms.

In conclusion, 6WM 4D Raman–electronic spectra were measured of the cyanine dye IR-140, free of unwanted lower-order cascaded signals. Use of a non-resonant pre-pump enables unambiguous assignment of coherences to either ground or excited electronic state pathways. This specificity can provide much needed evidence for distinguishing vibrational and electronic coherences in light harvesting protein complexes where discovering the origin of the observed coherent signatures may hold the key to better understanding their efficient energy transfer. Spatial encoding of time delays (as in GRAPES) enabled an otherwise intractable 4D measurement to be acquired. By isolating distinct vibronic pathways using simultaneous electronic and vibrational frequency resolution, GAMERS reveals sub-structure within 2D electronic peak shapes that is otherwise concealed under broadened electronic features. The resulting GAMERS spectra contain a wealth of vibrational and electronic coupling information which has heretofore been hidden by the lower dimensionality of 4WM techniques.

## Methods

### Sample preparation and handling

The sample was prepared by dissolving solid IR-140 (Sigma-Aldrich) in spectrophotometric grade methanol and passing the solution through a 0.45 μm filter, yielding a sample with an optical density of approximately 0.3 at 775 nm in a 200 μm cuvette. During the experiment, the sample was flowed through a 200 μm path length cuvette in a closed loop using a peristaltic pump to minimize repetitive excitation. The total sample volume was ∼20 ml, which is a large enough volume to prevent significant sample photo-degradation over the course of 12 h of continuous data collection.

### Spectroscopic methods

Our experiments utilize a second harmonic–pumped, noncollinear optical parametric amplifier (ORPHEUS-N 2H, Light Conversion), or NOPA, that produces 25 fs pulses with a centre wavelength of 775 nm and that is pumped by the 1,028 nm wavelength, 200 kHz repetition rate output of a Yb:KGW laser system (PHAROS, Light Conversion). The NOPA output is first spatially filtered and then routed into a passively phase-stable GRAPES apparatus (see Supplementary Fig. 7) where it is split evenly into three 34 nJ excitation pulses. Passive phase stabilization^40^ is achieved by routing the excitation (1, 2, and 3) and local-oscillator (4) pulses in a pairwise manner with common optics, wherein pulses 1 and 3 utilize a common delay line, as do pulses 2 and 4. In such an arrangement, phase fluctuations due to changes in delay between pulses 1 and 2 (*τ*) are cancelled by approximately equal and opposite fluctuations between pulses 3 and 4 (*t*); this cancellation becomes exact in the limit of zero signal bandwidth. To enable 6WM measurements, this GRAPES setup was modified to incorporate an additional non-resonant pre-pump, denoted as pulse 0, which is generated by a NOPA (ORPHEUS-N 3H, Light Conversion) that is pumped by the same Yb:KGW laser system described above. Pre-pump pulses have a centre wavelength of 850 nm, a pulse duration of 60 fs, and a pulse energy of 120 nJ. The beam geometry is set such that pulse 0 has the same wavefront tilt at the sample as pulse 1, ensuring that there is no spatial time delay gradient between pulses 0 and 1. All five pulses (0, 1, 2, 3, and 4) are focused to a ∼5 mm long vertical line in the sample using a 20 cm focal length cylindrical mirror.

The desired 6WM signal involves two oppositely-signed light-matter interactions with the pre-pump (pulse 0) followed by one interaction with each of the three pulses (1, 2, and 3) from the typical 4WM pulse sequence. Therefore, the 6WM signal is emitted in the phase-matched directions defined by 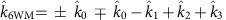. After the sample, the 6WM signal is spatially filtered from the excitation beams, re-imaged to the slit of a spectrograph (TRIAX 320, ISA), and acquired by a CMOS image sensor (Zyla 5.5 sCMOS 10-tap, Andor). In the partially-collinear beam geometry utilized here, 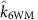 approximately coincides with the phase-matched direction for 4WM signal generated by pulses 1, 2 and 3 (given by 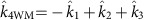), differing only in the additional spread in 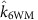 due to the nonzero bandwidth of pulse 0. Therefore, in order to isolate the 6WM signal from the collinear 4WM signal, a chopping and subtraction scheme is employed wherein pulses 0 and 3 are chopped at 100 Hz and 200 Hz, respectively. The 500th subharmonic (400 Hz) of the laser repetition rate is generated by a microcontroller (ATmega328, Atmel) and used to trigger the camera and choppers such that they are phase-locked to the laser pulse train. The state of both choppers was recorded for each image, allowing the 6WM signal to be isolated by adding sets of 4 sequential images according to 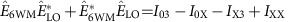 where 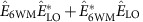 are the complex-valued 6WM–LO interference terms, *I*_03_ is the intensity image when both pulses 0 and 3 are unblocked, *I*_0X_ and *I*_X3_ are the intensity images when either pulse 3 or pulse 0 is blocked respectively, and *I*_XX_ is the intensity image when both pulses 0 and 3 are blocked.

Two-dimensional delays scans were acquired by scanning the delay between pulses 0 and 1 (*T*_0_) at a fixed *T* delay, and then repeating the *T*_0_ scan for a set of *T* delays. At each *T*, the *T*_0_ delay is scanned 5 times, with 1,200 spatial-spectral interferogram images acquired at each *T*_0_, ultimately totalling 6,000 images at each (*T*_0_, *T*) point. Following the chopper subtraction procedure, these 6,000 images are reduced to 1500 background-subtracted images which are subsequently averaged together. The resulting 4D data sets contain the 6WM signal as a function of four time–frequency dimensions: 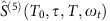.

### Data processing

To process the 2D delay scan data sets, the averaged images at each (*T*_0_, *T*) point are Fourier-filtered to isolate one of the two interference peaks between the 6WM signal and local oscillator. The spatial-interferometry pulse tilt correction^41^ is applied to the images to remove the phase introduced by the wavefront tilt between pulse 3 and the local oscillator (pulse 4), and the images are interpolated along the detection axis onto a grid of equally spaced frequency points. At this point, 6WM 2DFT electronic slice spectra can be generated. These spectra, 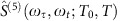, are a function of *ω*_*τ*_ and *ω*_*t*_ and depend parametrically on *T*_0_ and *T*.

To generate the 4D frequency spectrum of the 6WM signal, we first isolate the coherent signal components, which appear as damped oscillations along *T*_0_ and *T*, from other exponentially decaying ‘population-like' signal components. This is necessary in this case since the frequency spectrum of the strong exponentially decaying population signals partially overlaps that of the low-frequency coherent beats of interest here. Since the late (1 ps) *T*_0_ and *T* slices effectively capture the exponential background of the overall data set, they are subtracted from the 4D data set along the appropriate dimensions (that is, 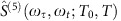 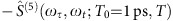 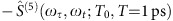) in order to remove the population signals without removing the coherent signals of interest. The remaining oscillatory components are apodized along *T*_0_ and *T* using a Tukey window and finally 2D Fourier-transformed, yielding the 4D electronic-Raman spectrum, 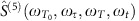.

### Data availability

The data that support the findings of this study are available from the corresponding author on reasonable request.

## Additional information

**How to cite this article:** Spencer, A. P. *et al*. Quantum coherence selective 2D Raman–2D electronic spectroscopy. *Nat. Commun.* **8,** 14732 doi: 10.1038/ncomms14732 (2017).

**Publisher's note**: Springer Nature remains neutral with regard to jurisdictional claims in published maps and institutional affiliations.

## Supplementary Material

Supplementary Information Supplementary Figures and Supplementary Notes
