# Supplementary material for: Quantum coherence selective 2D Raman–2D electronic spectroscopy
Source: Nat Commun. 2017 Mar 10;8:14732. doi: 10.1038/ncomms14732 (PMC5353627; doi:10.1038/ncomms14732)
Supplement: Supplementary Information — Supplementary Figures and Supplementary Notes [file ncomms14732-s1.pdf]

1. Supplementary Note 1: Tractability analysis for non–spatially multiplexed 4D 6WM measurements

Without the benefit of spatial multiplexing, as in GAMERS, 4D 6WM measurements would require scanning of three time dimensions: T_0, τ, and T. Using the same laser intensity as in GAMERS, sampling of the 150 τ data points would take approximately 15 times longer. This estimate comes from the larger number of data points scanned (150× more in the non-multiplexed case) which is somewhat offset by the greater integrated pulse energy used per detector pixel (10× assuming 65 µm tall pixels in the non-multiplexed case compared to 6.5 µm pixels in GAMERS). Since the spatially multiplexed experiment already requires 12 hours to complete a 2D scan of T_0 and T, the non-multiplexed experiment would take 7.5 days. Since phase drift within the apparatus must be maintained to no more than a small fraction of the wavelength of the signal field, this longer experimental duration places much stricter requirements on phase stability. If high phase stability is not realized, the signal-to-noise ratio of the resulting measurements will be significantly reduced compared to the multiplexed GAMERS experiment.

2. Supplementary Note 2: 6WM results for IR-895, IR-140, and IR-895 using a 1028 nm pre-pump

In addition to the measurements described in the main text that utilized a NOPA to generate the non-resonant pre-pump, 6WM measurements were also taken using 1028 nm, 220 fs, 2.6 µJ pre-pump pulses derived from a Yb:KGW laser amplifier. These measurements were carried out on solutions of IR-140, IR-895, and IR-144 in methanol and the results are shown in Supplementary Figures 1–6.

For each of the three dyes studied here, the 6WM-2DES spectra resemble the 4WM 2DFT electronic spectra at comparable T delays except for subtle dynamic peak shifts as a function of T_0 in IR-895 and IR-140. In IR-895, an initial ~100 cm^{-1} redshift along ω_τ (relative to the 4WM 2DFT spectrum) recedes as T_0 progresses beyond pulse overlap. Conversely in IR-140, an initial zero peak shift along ω_τ at early T_0 grows into a ~100 cm^{-1} redshift once T_0 is outside of pulse overlap.

The 6WM-2DBS spectra of IR-895, IR-140, and IR-144 contain similar features, yet exhibit differences in peak shapes and frequencies. Beating spectra of all three dye samples contain both a diagonal peak in the 140 cm^{-1} to 150 cm^{-1} region along with a lower frequency 65 cm^{-1} to 75 cm^{-1} diagonal peak. The limited temporal bandwidth of the pre-pump pulse is expected to attenuate the higher frequency beat by as much as a factor of 25, significantly altering its amplitude relative to the lower frequency beat. The higher frequency peak is centered at slightly different frequencies for each of the three dyes, ranging from 140 cm^{-1} in IR-895 to 150 cm^{-1} in IR-140 with IR-144 falling in between at 145 cm^{-1}. Likewise, the lower frequency peak location varies from 65 cm^{-1} in IR-895 and IR-144 to 75 cm^{-1} in IR-140. In addition to these diagonal features, cross peaks link some of the diagonal peaks to zero-frequency features along one or both dimensions. These cross peaks are located along the lines $\omega_{T_0} = 0$ or $\omega_T = 0$ and indicate coupling between population (zero-frequency) dynamics and coherent vibrational oscillations.

The beating signatures observed using the 1028 nm pre-pump are consistent with the results obtained with the 775 nm pre-pump when differences in pulse bandwidth are accounted for. The limited (sub-100 cm^{-1}) bandwidth of the 1028 nm pre-pump heavily attenuates the higher frequency features that are visible in the 775 nm pre-pump measurements. The remaining oscillatory features are somewhat distorted by a strong exponentially-decaying background signal that persists during the initial ~300 fs of T_0 due to pulse overlap. The combination of frequency-dependent attenuation and pulse overlap effects may result in peak shifts as large as 20 cm^{-1}.

3. Supplementary Note 3: Interpreting Energy Ladder Diagrams

In an energy ladder diagram (i.e., wave mixing diagram), the direction (upward versus downward) and style (solid [change in ket] versus dashed [change in bra]) of arrow determines the sign of the interaction frequency. Positive frequencies denote absorptive components and arise from either upward solid arrows or downward dashed arrows. Negative frequencies represent emissive components and involve either downward solid arrows or upward dashed arrows. Each arrow represents a light-matter interaction, with time running from left to right. The coherence frequency at any point in the diagram is equal to the difference frequency between the energy levels pointed to by the most recent (right-most) solid and dashed arrows (i.e., the sum of all prior interaction frequencies). The coherence frequency is positive when the ket (solid) is in a higher level than the bra and negative when the bra is in a higher level than the ket.

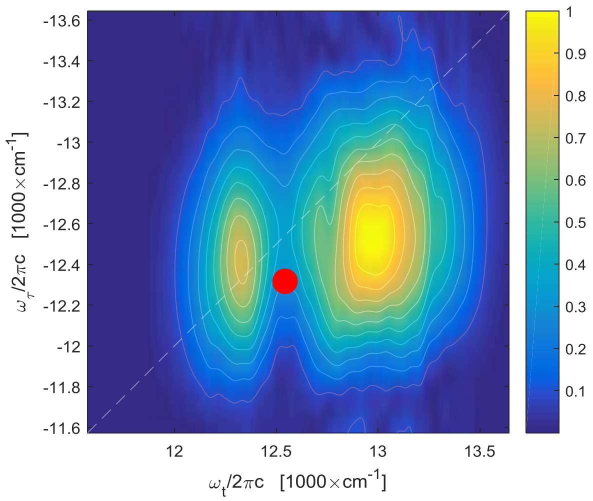

Supplementary Figure 1. Absolute value 6WM electronic spectrum of IR-895 at $T_0 = 250$ fs and $T = 100$ fs.

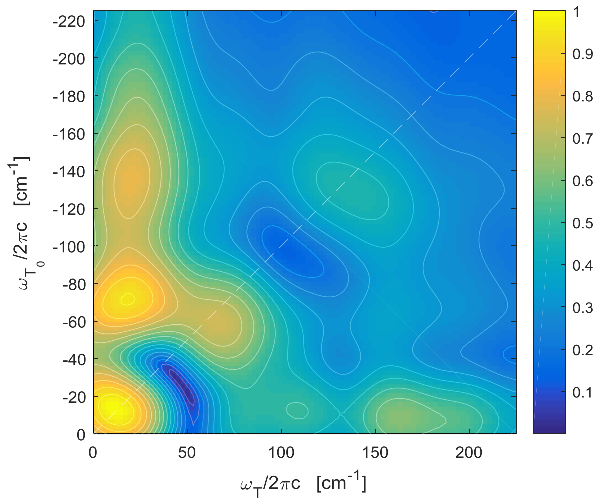

Supplementary Figure 2. Absolute value 6WM beating spectrum of IR-895 taken at the (ω_τ, ω_t) location indicated by the red dot in Supplementary Figure 1.

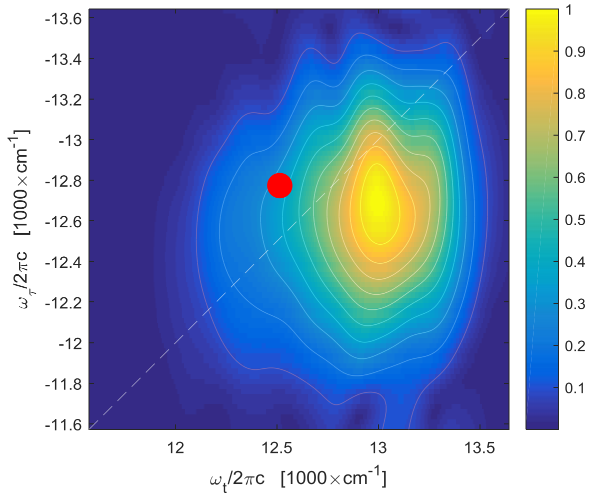

Supplementary Figure 3. Absolute value 6WM electronic spectrum of IR-140 at $T_0 = 250$ fs and $T = 100$ fs.

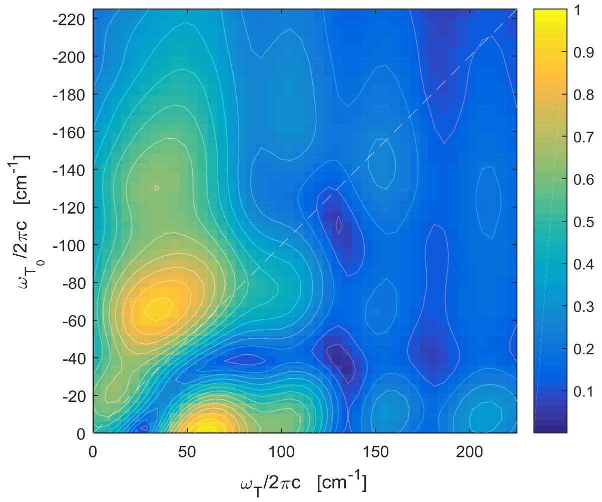

Supplementary Figure 4. Absolute value 6WM beating spectrum of IR-140 taken at the (ω_τ, ω_t) location indicated by the red dot in Supplementary Figure 3.

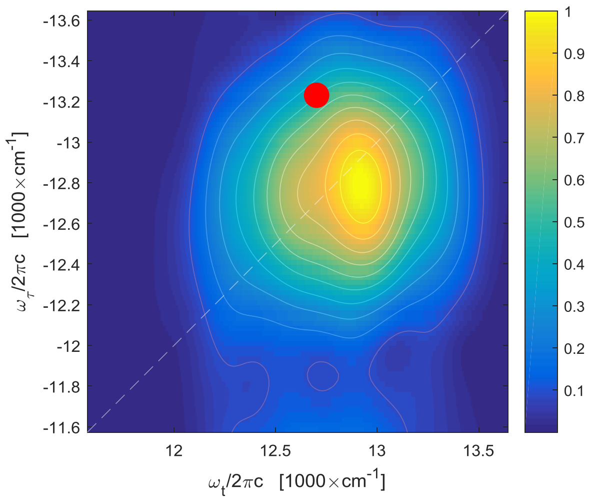

Supplementary Figure 5. Absolute value 6WM electronic spectrum of IR-144 at $T_0 = 250$ fs and $T = 100$ fs.

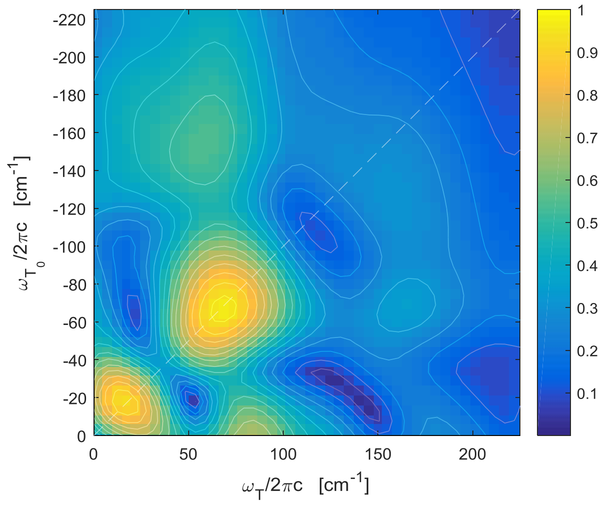

Supplementary Figure 6. Absolute value 6WM beating spectrum of IR-144 taken at the (ω_τ, ω_t) location indicated by the red dot in Supplementary Figure 5.

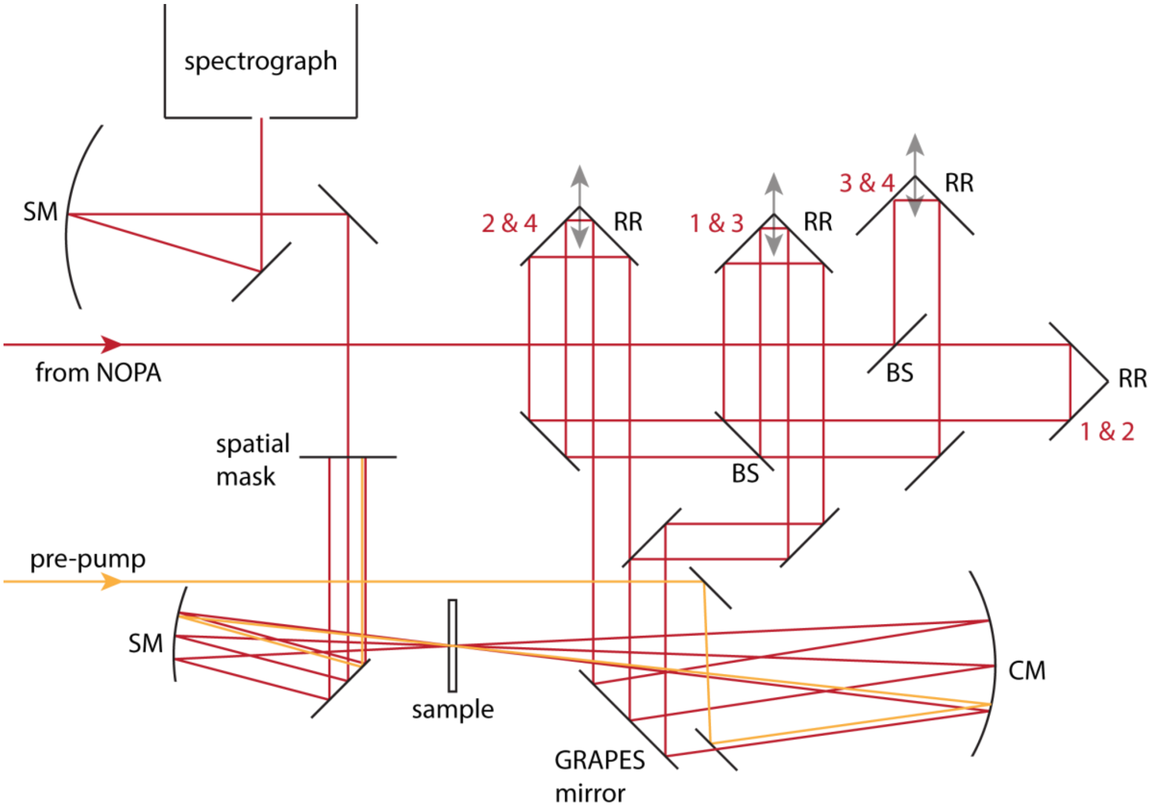

Supplementary Figure 7. Schematic of the GAMERS experiment. Labeled optics include retroreflectors (RR), beam splitters (BS), a cylindrical mirror (CM), and spherical mirrors (SM). Unlabeled optics are flat protected silver mirrors. Motorized delay stages are indicated by arrows. Delay stages are labeled based on which beams they delay. The GRAPES mirror is composed of 4 independently adjustable mirrors, enabling pulse tilts to be applied to beams 1 and 4 such that all excitation beams overlaps spatially at the focus in the sample.
